# Neuronal Expression of the Human Neuropeptide S Receptor NPSR1 Identifies NPS-Induced Calcium Signaling Pathways

**DOI:** 10.1371/journal.pone.0117319

**Published:** 2015-02-25

**Authors:** Frank Erdmann, Sebastian Kügler, Peter Blaesse, Maren D. Lange, Boris V. Skryabin, Hans-Christian Pape, Kay Jüngling

**Affiliations:** 1 Institute of Physiology I, Neurophysiology, Westfälische Wilhelms-University Münster, Robert-Koch-Strasse, 27a, 48149, Münster, Germany; 2 Center of Molecular Physiology of the Brain (CMPB), Department of Neurology, University Medicine Göttingen, Waldweg, 33, 37073, Göttingen, Germany; 3 Institute of Experimental Pathology, ZMBE and Interdisciplinary Clinical Research Center, Westfälische Wilhelms-University Münster, Von-Esmarch-Str. 56, 48149, Münster, Germany; School of Medicine and Health Sciences, University of North Dakota, UNITED STATES

## Abstract

The neuropeptide S (NPS) system was discovered as a novel neurotransmitter system a decade ago and has since been identified as a key player in the modulation of fear and anxiety. Genetic variations of the human NPS receptor (NPSR1) have been associated with pathologies like panic disorders. However, details on the molecular fundamentals of NPSR1 activity in neurons remained elusive. We expressed NPSR1 in primary hippocampal cultures. Using single-cell calcium imaging we found that NPSR1 stimulation induced calcium mobilization from the endoplasmic reticulum via activation of IP_3_ and ryanodine receptors. Store-operated calcium channels were activated in a downstream process mediating entry of extracellular calcium. We provide the first detailed analysis of NPSR1 activity and the underlying intracellular pathways with respect to calcium mobilization in neurons.

## Introduction

In 2004, the 20 amino acid neuropeptide S (NPS) has been identified as the ligand for the formerly orphan g-protein coupled receptor GPR-154 (now NPS receptor, NPSR1) [1], with NPS/NPSR1 forming a novel neurotransmitter system in the brain [2]. NPS is expressed in the brainstem and in endocrine tissue of rodents, while NPSR1 mRNA can be detected in various brain regions, mainly in the cortex, thalamus, hypothalamus, and in the amygdala [1,3].

Since its discovery, the NPS system has been found to modulate behavior in rodents, mediating anxiolytic effects [1,4–8], stimulating locomotion [1,6,9–11], increasing arousal [1,12], and decreasing food intake [13,14]. Human studies have linked the NPS system and single-nucleotide polymorphisms in the *NPSR1* gene to diseases like asthma and allergy [15,16], inflammatory bowel disease [17], rheumatoid arthritis [18] and panic disorders [19–22].

Despite these numerous biological functions, little is known about the molecular mechanisms underlying NPS receptor activity. Application of NPS in HEK293 or CHO cells expressing human NPSR1 variants has been found to elicit calcium mobilization and cAMP accumulation [1,10,23–25], indicating that the receptor activates Gα_q_ and Gα_s_ pathways, respectively. In Colo205 cells expressing NPSR1, NPS leads to dose-dependent stimulation of cell proliferation and MAP kinase phosphorylation [24]. However, the intracellular pathways downstream to NPSR1 activation have not been identified in detail.

In the present study we aimed to identify intracellular pathways activated following NPSR1 stimulation in neurons. The focus was on the mechanisms of intracellular calcium mobilization, in view of previous results obtained with NPSR1 stimulation in non-neuronal cells [1,24]. Rather than using stable transfected model cell lines, we made use of an adenoviral system to express NPSR1 in cultured mouse hippocampal neurons, combined with confocal calcium imaging and pharmacology. Through this experimental design, we provide the first detailed characterization of NPSR1 function and intracellular signaling pathways in neurons.

## Materials and Methods

### Ethics statement

All experiments were carried out in accordance with the European Committees Council Directive (86/609/EEC) for experimentation on animals. Protocols were approved by the Landesamt für Natur, Umwelt-und Verbraucherschutz NRW (AZ 8.87–51.05.20.10.114).

### Vector production

**Fig 1:**
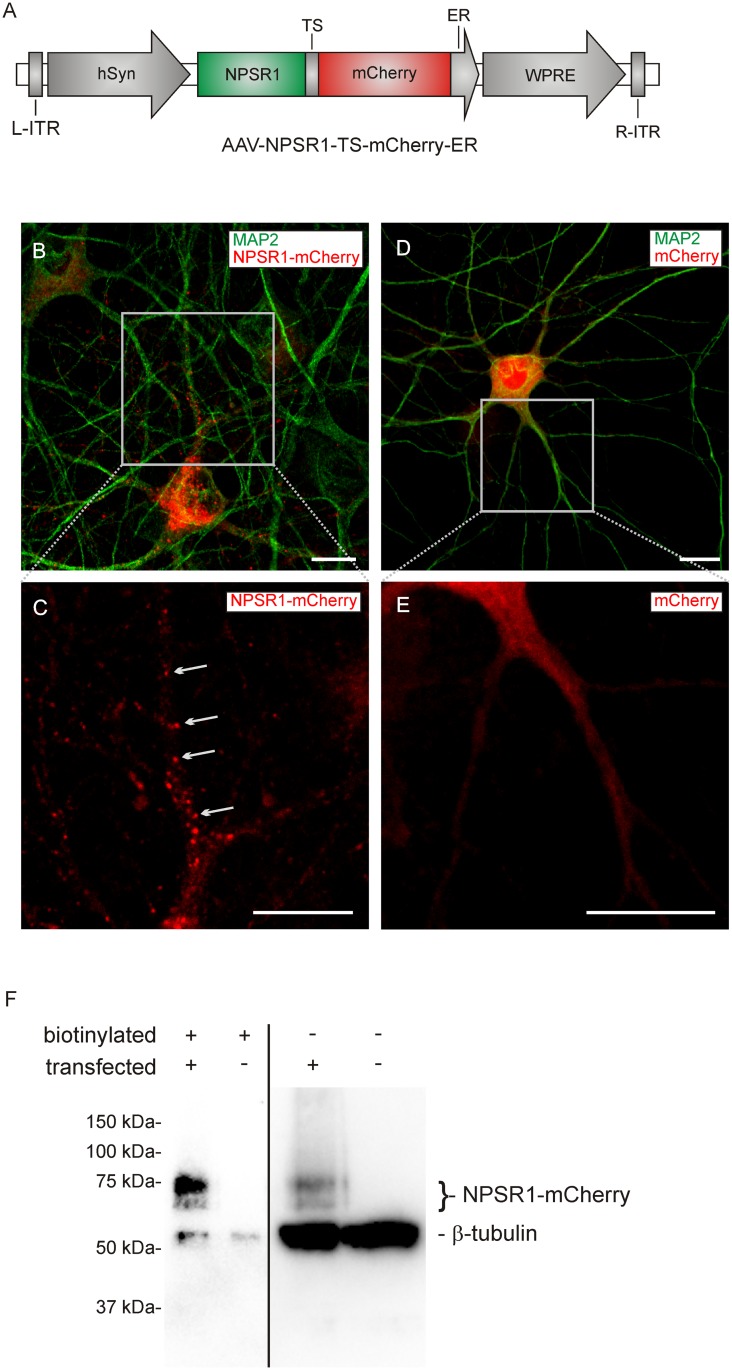
Vector design and expression of NPSR1. (A) Construct design of the AAV-NPSR1-TS-mCherry-ER vector. NPSR1 was expressed fused to mCherry. A plasma membrane targeting signal (TS) and an ER-export signal (ER) were used to enhance correct trafficking. (B,C) Immunocytochemistry of cultured hippocampus neurons, 9 days after plating and 7 days after viral transfection with AAV-NPSR1-TS-mCherry-ER. Green: MAP2-immunoreactivity (-IR), red: mCherry-IR. Punctual localization of NPSR1-mCherry is marked in (C). (D,E) Immunocytochemistry of cultured hippocampus neurons, 9 days after plating and 7 days after viral transfection with a control vector expressing mCherry but not NPSR1. Punctual localization of mCherry could not be detected. Scale bars indicate 10 μm. (F) Surface expression of NPSR1-mCherry. Immunoblot with anti-mCherry and anti-neuronal class III β-tubulin for the biotinylated plasma membrane fraction of proteins and the non-biotinylated intracellular fraction. Biotinylation of cultured hippocampus neurons was performed 6 days after transfection with AAV-NPSR1-TS-mCherry-ER and untransfected neurons were used as control. Exposure times were 4 s for the biotinylated fractions and 0.45 s for the unlabeled fractions.

For viral transfection, DNA containing the coding sequences of the isoform A of the human neuropeptide S receptor (NPSR1, the amino acid sequence can be accessed through NCBI protein database under NCB accession # NP_997055), a plasma membrane targeting signal (TS), the red fluorescent protein mCherry and an ER export signal (ER) was commercially synthesized (Genscript, USA). This fragment was subcloned into an AAV vector containing ITRs of serotype 2, the human synapsin promoter (hSyn), the woodchuck hepatitis virus posttranscriptional regulatory element and a polyA site resulting in AAV-NPSR1-TS-mCherry-ER (Fig. 1A). For control experiments, an AAV expressing mCherry under the control of hSyn was used (AAV-mCherry). This vector contained ITRs of serotype 2, coding sequences for the human synapsin promoter, a 2A sequence, the red fluorescent protein mCherry, the woodchuck hepatitis virus posttranscriptional regulatory element, and a polyA site. To express NPSR1 as a non-fusion protein, the human synapsin promoter, the coding sequences for a hemagglutinin signal sequence, NPSR1, an IRES sequence, and the red fluorescent protein tdTomato were cloned into a pcDNA3.1 vector (NPSR1-IRES-tdTomato, Invitrogen, USA).

### Virus production

Recombinant adeno-associated virus particles of serotype 6 (AAV6) were produced essentially as described [26]. AAV-mCherry was a kind gift from Dr. Stephan Guggenhuber and Prof. Dr. Beat Lutz (Institute of Physiological Chemistry, University Medical Center of the Johannes Gutenberg University Mainz, Mainz, Germany).

### Animals

Animals were kept in temperature-controlled, air-filtered cages with 12h light-dark-cycle and unlimited access to water and food. Animals were controlled for signs of distress on a daily basis.

### Cell culture and transfection

P0–P2 C57BL/6 mice were sacrificed without anesthesia by decapitation. Hippocampal cultures were prepared as described elsewhere [27]. In brief, hippocampi were dissected and trypsinated (0.25%, Invitrogen, USA) for 15 min. Cells were triturated with fire-polished pasteur pipettes and plated on poly-D-lysine-coated coverslips (1 mg/ml, Invitrogen) in the presence of AraC (25 μM, Invitrogen). After 24 h, Eagle’s basal medium (BME, Invitrogen) was exchanged to Neurobasal (Invitrogen), complemented with B27, Glutamax (Invitrogen), 1% fetal calf serum and 1% penicillin/streptomycin. 24–48 h after seeding, AAV-NPSR1-TS-mCherry-ER was added to the medium (4 x 10^6^ transducing units in 2 ml). Half of the medium was replaced by fresh Neurobasal every 3–4 days. Transfection of the NPSR1-IRES-tdTomato vector was done using Lipofectamin 2000 (Invitrogen) in serum-free Neurobasal following manufacturer recommendations. In brief, 1 μg vector DNA and 2 μl LF2000 were used per coverslip and incubated for 90 min. Neurons were transfected after 7 days *in vitro*.

### Surface biotinylation

Surface biotinylation was performed as described elsewhere [28]. 6 days after transfection with AAV-NPSR1-TS-mCherry-ER, hippocampal neurons were labeled with biotin (100 μM, Sigma-Aldrich, USA) in PBS on ice for 30 min. Cells were washed in 1 M glycine in PBS on ice for 10 min and homogenized in RIPA buffer (150 mM NaCl, 1% Triton X-100, 0.5% deoxycholic acid, 0.1% SDS, 50 mM Tris-Cl pH 8.0) supplemented with a protease inhibitor mixture (Complete Mini EDTA free protease inhibitor mixture, Roche). Biotinylated proteins were purified with 50 μl streptavidin agarose beads (Sigma-Aldrich) in RIPA buffer at 4°C overnight and eluted in SDS-PAGE sample buffer containing 5% β-mercaptoethanol at room temperature (RT).

### SDS-PAGE and western blotting

The complete eluent from the agarose beads (membrane fraction) and 29% of the supernatant (unlabeled intracellular proteins) were separated in a 10% SDS-PAGE and subsequently blotted on nitrocellulose membranes (Perkin Elmer) in transfer buffer (25 mM Tris, 192 mM glycine, 10% methanol, pH 8.3). Membranes were blocked for 1h at RT in TBS-T (20 mM Tris, 150 mM NaCl, 0.1% Tween-20, pH 7.5) containing 4% non-fat dry milk (Fluka). Membranes were incubated with primary antibodies (rabbit anti-mCherry, 1 mg/ml, 1:1,000, ab167453, Abcam and rabbit anti-neuronal class III β-tubulin, 1 mg/ml, 1: 40,000, PRB-435P, Covance) at 4°C overnight in blocking buffer. After washing, the secondary antibody (horseradish peroxidase-coupled goat anti-rabbit immunglobulins, 1:2,000, DAKO) was applied for 1h at RT in TBS-T/4% milk. After washing, immunosignals were detected using a supersignal western blot detection kit (Thermo Scientific) and a Chemidoc MP imaging system (Biorad). Exposure time was 4 s for the biotinylated fraction and 0.45 s for the unlabeled fraction. Immunoblots were analysed using the software ImageJ [29].

### Calcium imaging

If not stated otherwise, calcium imaging experiments were performed with extracellular solution containing 125 mM NaCl, 2.5 mM KCl, 1.25 mM NaH_2_PO_4_, 30 mM HEPES, 10 mM Glucose, 3 mM CaCl_2_, 2 mM MgSO_4_, NaOH pH 7.35. For calcium-free measurements, CaCl_2_ was substituted by MgCl_2_ and 5 mM EGTA was added. Solutions were supplemented with 125 nM tetrodotoxin (TTX, Tocris, UK). 5 to 7 days (AAV) or 2 to 3 days (Lipofectamin2000) after transfection, cells were stained with 2 μM Fluo4-AM (Molecular probes, USA) in extracellular solution at RT for 23 min. Cells were continuously perfused (13 ml/min) with extracellular solution at RT and objected to imaging using the 488 nm line of a Nikon eC1 plus laser scanning confocal microscope (Nikon, Japan). A Nikon 16x/0.80w water immersion objective was used. Images were acquired every 2.5 sec using the software suite Nikon EZ-C1. NPS was custom-synthesized by Genscript (USA). Stock solutions were prepared in PBS and diluted to final concentrations with extracellular solution. As a general rule, cells were stimulated for 90 s with 500 nM NPS if not indicated otherwise. Experiments with CPA, 2-APB, U73122, ryanodine and SHA68 started with the application of NPS to identify responsive neurons. NPS was allowed to wash out for at least 10 min, followed by drug application. NPS was applied once again to test for drug effects on NPSR1 activity. For reversible drugs, NPS was finally applied for a third time after drug wash out. Incubation times prior to NPS application were 300 s for 2-APB, U73122, ryanodine and SKF96365, 180 s for SHA68, 120 s for ML-9 and 600 s for the Ca_v_ blocker cocktail. Washout times were 900 s for 2-APB and SHA68. Neurons were incubated with CPA for 500–650 s, until fluorescence reached baseline level. Blockers were used in the following concentrations: 30 μM CPA (Tocris), 25 μM 2-APB (Sigma-Aldrich), 10 μM U73122 (Tocris), 50 μM ryanodine (Tocris), 1 μM SHA68 [10], 50–100 μM ML-9 (Tocris) and 15 μM SKF96365 (Tocris). The cocktail used to block Ca_v_s contained 4 μM mibefradil, 5 μM nifedipine and 250 nM conotoxin MVIIC (all Tocris). For calcium-free measurements, neurons were incubated with calcium-free solution for 50 s prior to NPS-application. In order to discriminate neurons from other cell types, 300 μM glutamate (Tocris) was applied for 1 s at the beginning or end of each experiment.

### Immunocytochemistry

Cultured neurons were fixed in ice-cold 4% paraformaldehyde for 20 min and incubated in 10% normal goat-serum, 1% BSA, 0.3% Saponine (blocking solution) in PBS for 1 hour at RT. Primary antibodies (mouse anti-mCherry, 1 mg/ml, 1:200, orb66657, Biorbyt, UK, guinea pig anti-MAP2, 1 mg/ml, 1:1000, 188004, Synaptic Systems, Germany) were diluted in blocking solution. Cells were incubated over night at 4°C. Secondary antibodies (goat anti-mouse Alexa594, 2 mg/ml, 1:1000, A11032, Invitrogen, donkey anti-guinea pig Alexa488, 1 mg/ml, 1:1000, 706545148, Jackson Immuno Research, UK) were diluted in blocking solution, and cells were incubated for 90 minutes at RT. Stained cells were mounted using Immu-Mount (Fisher Scientific, USA) and analyzed with a laser scanning confocal microscope (Nikon eC1 plus, Nikon, USA) using a Leica 40x/1.25–0.75 HCX APO OIL objective. Z-stacks (7 to 10 Z-frames, 0.5–1 μm/step) were scanned with a resolution of 1024x1024 pixels.

### Data analysis

The software ImageJ [29] and Excel 2010 (Microsoft, USA) was used for analyzing calcium imaging data. ROIs were applied to the soma of all glutamate-responsive neurons, defined by an increase of fluo-4 fluorescence intensity of the soma of at least 5x over baseline upon glutamate application. Intensity values from individual ROIs were normalized to baseline conditions prior to substance application. Calcium signals of individual cells were peak-aligned using Clampfit10 (Molecular Devices Corporation, USA). Fluorescence traces were averaged and plotted with Origin 9.0 (OriginLab, USA). Fluorescence amplitudes were calculated referring to baseline conditions. Amplitude values for baseline conditions represent maximum values within the baseline referring to the mean values of the baseline. Integration of the response amplitude over time for slow component of the calcium signal was done in Clampfit10. Image sequences from immunocytochemistry were volume rendered with EZ-C1 Viewer (Nikon). Prism (Graphpad, USA) was used for statistical analysis. One-way ANOVA with Tukey post-hoc tests or student’s t-tests were used as applicable. P values for Tukey post-hoc tests are given as multiplicity adjusted p values. Asterisks in figures indicate significant differences in the tested datasets (* = p < 0.05, ** = p < 0.01, *** = p < 0.001). Data are presented as mean with standard deviation if not indicated otherwise. All figures were prepared for presentation using CorelDraw Graphics Suite 12 (Corel Corporation, USA).

## Results

### Vector design and expression of NPSR1 in mouse neurons

In a first set of experiments we developed a protocol for the functional expression of NPSR1 in cultured mouse hippocampus neurons. We designed an adeno-associated viral expression vector (AAV-NPSR1-TS-mCherry-ER, Fig. 1A) that contained the coding sequences for NPSR1 and mCherry, separated by a plasma membrane targeting signal (KSRITSEGEYIPLDQIDINV), and a C-terminal ER-export signal (FCYENEV), both derived from isoforms of an inward-rectifying potassium channel (Kir2.1/Kir2.4) [30]. These signal peptides enhance trafficking along the secretory pathway in heterologous expression systems, a strategy successfully applied for the neuronal expression of microbial opsins [31]. With this construct, NPSR1-mCherry positive neurons could be detected by red fluorescence from 4 days after transfection (Fig. 1B,C). We found punctual clusters of NPSR1-mCherry in the somatic region and along dendritic structures as indicated by the immunoreactivity (IR) of the dendritic marker MAP2 (Fig. 1B,C). In contrast, mCherry alone did not show a punctual localization, but was evenly distributed within the cell (Fig. 1D,E). To quantify the transfection efficiency, neurons were selected by their MAP2-IR 5–7 days after incubation with AAV-NPSR1-TS-mCherry-ER (see materials and methods). In 95 ± 7% of MAP2-positive neurons, the red fluorescence of NPSR1-mCherry could be detected (n = 230 neurons). Next, the membrane localization of the NPSR1-mCherry fusion protein was tested. We used surface biotinylation and subsequent immunoblotting to distinguish proteins located in the plasma membrane from the intracellular pool of proteins (Fig. 1F). As expected and demonstrating the specificity of the surface labeling assay, the signal intensity of the intracellular protein β-tubulin was high in the intracellular fraction, but very low in the membrane fraction. While a quantitative comparison of the two subcellular fractions is difficult due to the lack of a common loading control, the strong NPSR1 signal in the plasma membrane fraction as well as the difference in the NPSR1/β-tubulin ratios of the plasma membrane fraction (high ratio) and the intracellular fraction (low ratio), provided strong evidence for plasma membrane insertion of NPSR1-mCherry. NPSR1 was neither detectable in the plasma membrane nor in the intracellular fraction prepared from untransfected controls. Interestingly, NPSR1-mCherry showed up in two bands (74 kDa and 67 kDa) in the plasma membrane as well as in the intracellular fraction (Fig. 1F). The calculated molecular weight (MW) of the fusion protein is 73.7 kDa.

### Activation of NPSR1 in hippocampus neurons leads to calcium-release from intracellular stores

**Fig 2:**
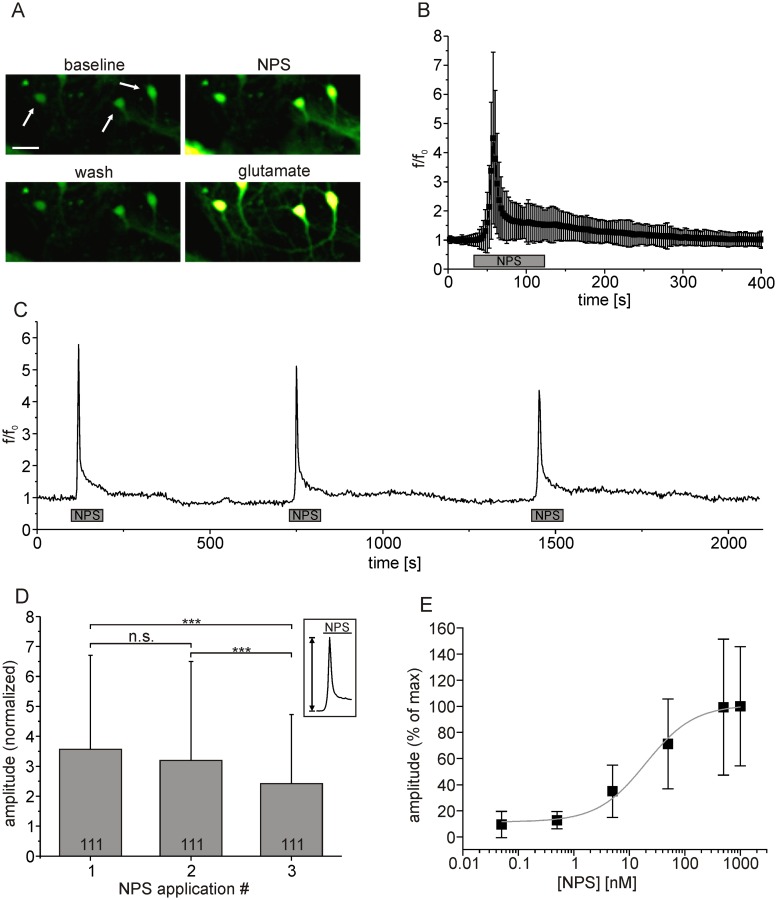
NPS-evoked calcium signals in hippocampus neurons expressing NPSR1. (A) Mean fluorescence images of Fluo4-stained hippocampus neurons transfected with AAV-NPSR1-TS-mCherry-ER during baseline-conditions, application of 500 nM NPS, washout and application of 300 μM glutamate. Arrows mark NPS-responsive neurons. Mean fluorescence images were calculated from seven raw images each. Scale bar indicates 25 μm. (B) Normalized mean fluorescence values for the first NPS-administration calculated from 229 individual neurons after peak alignment. (C) Fluorescence trace of a single NPSR1-expressing neuron stained with Fluo4-AM. NPS [500 nM] was applied for 90 seconds as indicated. (D) Comparison of normalized peak amplitudes for three consecutive applications of NPS. (E) Dose-response curve for NPSR1-mediated calcium mobilization in neurons, depicting an EC_50_ of 19.8 ± 1.3 (SEM, n = 318 neurons).

In view of previous findings in non-neuronal cell lines [1,24], we hypothesized that stimulation of NPSR1-expressing mouse hippocampus neurons with NPS induces an elevation of the cytosolic calcium concentration ([Ca^2+^]_cyt_). After transfection, cell cultures were incubated with Fluo4-AM and subjected to calcium imaging. Application of NPS resulted in a transient rise in Fluo4 fluorescence (Fig. 2A). In 229 neurons, the normalized mean fluorescence (f/f_0_) increased by 3.5 ± 2.9 over baseline (Fig. 2B). The time course of NPSR1-dependent calcium signals was characterized by an initial rapid component with a rise time of 9.47 ± 5.77 s (5–95% of max) followed by a slower recovery phase with a decay time (95–5% of max) of 132.71 ± 94.11 s. Repeated application of NPS resulted in a gradual decrease in response amplitude, measured as normalized mean fluorescence amplitude from 3.6 ± 3.1 for the 1^st^ application to 3.2 ± 3.3 (2^nd^) and 2.4 ± 2.3 (3^rd^) in 111 neurons (675 s interval, Fig. 2C,D, ANOVA: F_(2, 330)_ = 4.379, p = 0.0133). Application of varying NPS concentrations in a range of 50 pM to 1 μM resulted in a dose-response curve with an EC_50_ of 19.8 ± 1.3 nM (SEM, n = 318 cells, Fig. 2E).

**Fig 3:**
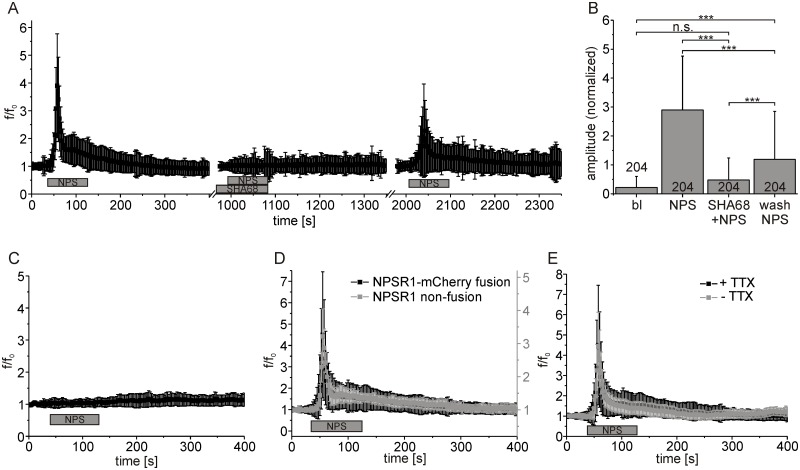
NPS-dependent calcium signals are abolished by the NPSR1 antagonist SHA68. (A) Mean fluorescence values recorded from 397 hippocampus neurons. NPS [250 nM] and the NPSR1 blocker SHA68 [1 μM] were applied as indicated. (B) Statistical comparison of mean fluorescence amplitudes from experiments shown in (A). SHA68 reduced the effect of NPS to a level non-significantly different from baseline (bl). (C-E) Control experiments. (C) In 936 untransfected hippocampus neurons, no increase in normalized mean fluorescence intensity could be detected when NPS was applied. (D) Normalized mean fluorescence values from 229 neurons expressing NPSR1 as a mCherry fusion protein from AAV-NPSR1-TS-mCherry-ER (black) and from 5 neurons expressing NPSR1 as non-fusion protein from NPSR1-IRES-tdTomato (grey). Please refer to the y-scale at the right-hand side (grey) for results from experiments with the non-fusion NPSR1. (E) Normalized mean fluorescence values from neurons expressing NPSR1 recorded in presence (n = 229, black, see Fig. 2B) or absence (n = 122, grey) of 125 nM TTX. NPS was used at 500 nM in the experiments,. if not indicated otherwise.

It has been shown that the NPSR1 antagonist SHA68 blocks NPS-mediated calcium mobilization in HEK293 cells expressing NPSR1 [10]. Here we used SHA68 to verify that recorded Ca^2+^ signals resulted from activation of NPSR1. In 204 neurons, application of NPS [250 nM] resulted in an increase in Fluo4 fluorescence with normalized mean amplitude of 2.9 ± 1.8 (Fig. 3A). In the presence of SHA68 [1 μM], NPS application failed to induce Ca^2+^ signals in the same neurons. The normalized mean amplitude was reduced to 0.5 ± 0.7, a level not significantly different from baseline (Tukey post- hoc test, p = 0.1815). After washout of SHA68, NPSR1 activity was partly restored, as indicated by NPS-evoked calcium responses with a normalized mean amplitude of 1.2 ± 1.6 (Fig. 3B, ANOVA: F_(3, 812)_ = 170.5, p < 0.0001).

In further control experiments, calcium responses could not be detected in 936 untransfected neurons after stimulation with NPS (Fig. 3C). Moreover, we tested if the C-terminal fusion of mCherry changes NPSR1-mediated calcium signals. When NPSR1 was expressed from a bicistronic vector as non-fusion protein, the evoked signals displayed a similar time course but reduced amplitude compared to the NPSR1-mCherry construct (Fig. 3D). In addition, NPSR1-mediated calcium signals were not affected when action potentials were suppressed by TTX [125 nM] (Fig. 3E).

**Fig 4:**
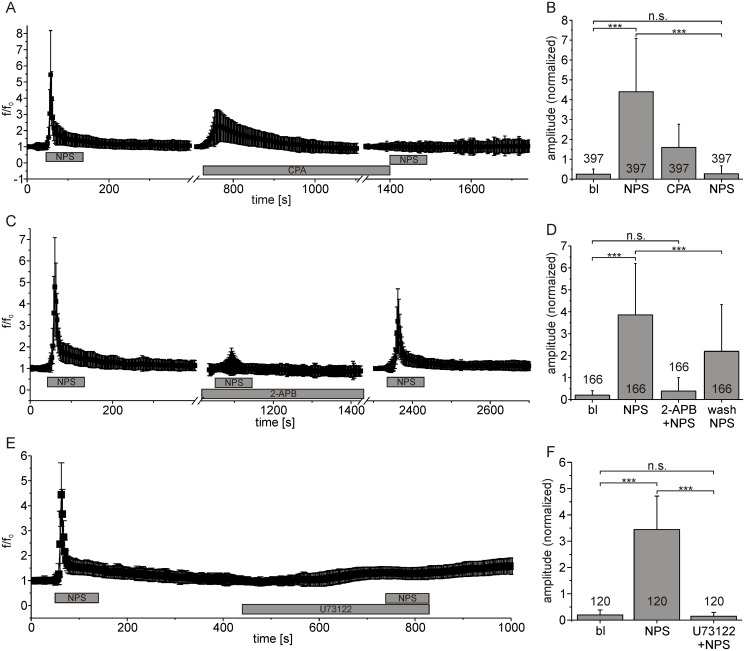
Calcium is released from the endoplasmic reticulum via IP_3_ receptors upon NPSR1 activation. (A) Normalized mean fluorescence calculated from 397 neurons. NPS and CPA [30 μM] were applied as indicated. CPA abolished NPS-evoked calcium signals. (B) Statistical analysis of peak amplitudes for baseline conditions (bl), NPS, CPA and NPS. (C) Mean fluorescence values from 166 cells treated with NPS and the IP_3_ receptor antagonist 2-APB [25 μM] as indicated. 2-APB abolished NPSR1-mediated calcium signals. (D) Peak amplitudes for baseline conditions, application of NPS, NPS in presence of 2-APB, and NPS after 2-APB washout calculated from the dataset shown in (C). (E) Mean fluorescence values from 120 cells treated with NPS and the PLC-blocker U73122 [10 μM]. The drug abolished NPSR1-mediated calcium signals. (F) Peak amplitudes for baseline conditions, application of NPS, and NPS in presence of U73122 calculated from the dataset shown in (E). NPS was used at 500 nM in all experiments.

To identify the source of calcium leading to an increase in [Ca^2+^]_cyt_ upon NPSR1 stimulation, we used cyclopiaconic acid (CPA) to inhibit SERCA-ATPases [32]. Under resting conditions, the concentration of free calcium in the cytosol is low while the concentration of free Ca^2+^ in the ER lumen is high. This is due to the action of SERCA, which constantly compensates for passive Ca^2+^ efflux from the ER (“calcium leakage”). In 397 neurons displaying intracellular calcium responses to NPS (mean fluorescence amplitude: 4.4 ± 2.69), subsequent application of CPA [30μM] resulted in a transient increase in cytosolic calcium (1.6 ± 1.17), indicating calcium leakage from the ER upon blockade of SERCA (Fig. 4A). After decline of CPA-evoked calcium transients, i.e. the ER was devoid of calcium, application of NPS failed to evoke intracellular calcium responses with a normalized mean amplitude non-significantly different from baseline (0.27 ± 0.4, Tukey post-hoc test, p = 0.9992). CPA abolished NPSR1-mediated calcium signals in NPS-responsive neurons (Fig. 4B, n = 397, ANOVA: F_(3, 1584)_ = 684.6, p < 0.0001).

These results let us hypothesize that calcium was released from the ER following NPSR1 stimulation. As NPSR1 has been described to stimulate the Gα_q_ pathway [1,24,25] which couples to inositol 1,4,5-trisphosphate receptors (IP_3_R) via phospholipase C (PLC)-mediated generation of IP_3_, we tested the involvement of IP_3_Rs and PLC in a next series of experiments. Application of the IP_3_R antagonist 2-APB [33] [25 μM] prevented responses to NPS (Fig. 4C). In 166 neurons, the normalized mean fluorescence amplitude evoked by NPS was reduced from 3.86 ± 2.34 for a first application to 0.39 ± 0.61 when NPS was applied in the presence of 2-APB, a level that was not significantly different from baseline (Tukey post-hoc test, p = 0.6122). The effect was reversible, with a normalized mean fluorescence amplitude of 2.2 ± 2.13 after 2-APB washout (Fig. 4D, ANOVA: F_(3, 660)_ = 239.8, p < 0.0001). We used the PLC inhibitor U73122 [34] [10μM] to block the generation of IP_3_ upon NPSR1 stimulation. In 120 neurons, the drug abolished NPS-mediated calcium release (Fig. 4E). The mean fluorescence amplitude was reduced from 3.44 ± 1.27 for a first application of NPS to 0.14 ± 0.14 when U73122 and NPS were co-applied, a level not significantly different from baseline (Tukey post-hoc test, p = 0.8935, Fig. 4F, ANOVA: F_(2, 357)_ = 758.3, p < 0.0001).

**Fig 5:**
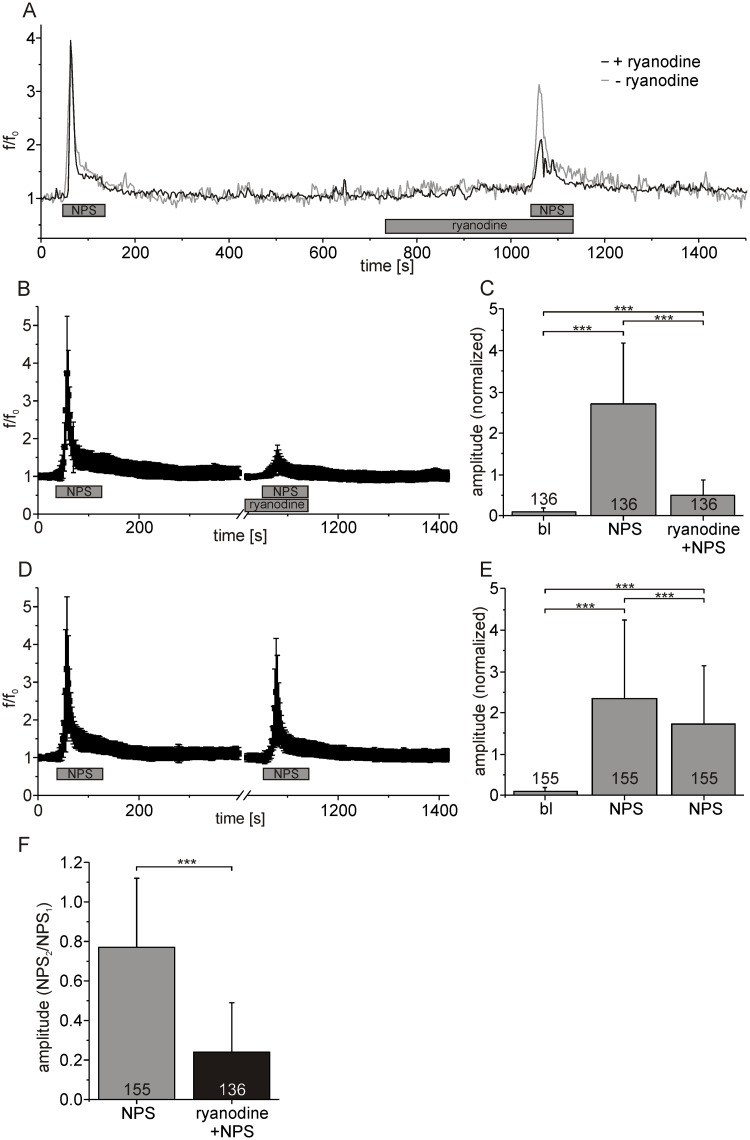
NPSR1-mediated IP_3_ receptor activation triggers calcium-induced calcium release via ryanodine receptors. (A) Fluorescence recording from a single neuron treated with ryanodine [50 μM] (black) and an untreated control (grey). (B) Mean fluorescence intensities calculated from 136 individual neurons. (C) Mean amplitudes calculated from the dataset shown in (B). (D) Fluorescence as averaged from 155 control neurons for two consecutive NPS applications in the absence of ryanodine. (E) Mean amplitudes calculated from the dataset shown in (D). (F) Mean amplitudes for the second application of NPS normalized to the preceding first NPS-administration in control cells (grey) and in the presence of ryanodine (black). NPS was used at 500 nM in all experiments.

Next, the possible contribution of ryanodine receptors (RyR) to NPSR1-mediated Ca^2+^ release was tested. In the presence of ryanodine [50 μM], the normalized mean fluorescence amplitude evoked by NPS was at 0.51 ± 0.37, a significant reduction compared to responses prior to ryanodine which were at 2.7 ± 1.48 (Fig. 5A-C, ANOVA: F_(2, 405)_ = 337.0, p < 0.0001). Control recordings from the same batch of neurons without ryanodine treatment showed that the normalized mean fluorescence amplitude was reduced from 2.35 ± 1.9 for a first application of NPS to 1.74 ± 1.4 when NPS was applied again (Fig. 5D,E, ANOVA: F_(2, 462)_ = 111.4, p < 0.0001). Taken together, the amplitude of the response evoked by a second NPS application reached 0.77 ± 0.35 when normalized to the first application, while this value was reduced to 0.24 ± 0.25 when RyRs were blocked (Fig. 5F, t-test: p < 0.0001).

### NPSR1 activation induces store-operated calcium entry

**Fig 6:**
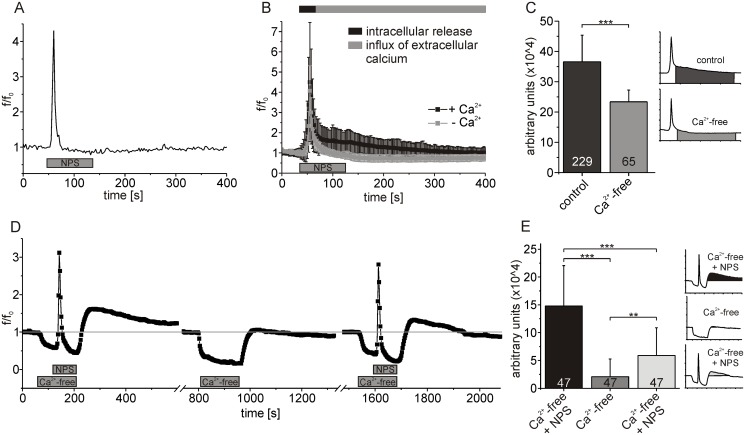
NPS application triggers store-operated calcium entry (SOCE) in neurons expressing NPSR1. (A) Single-cell fluorescence recording performed in calcium-free solution. (B) Mean fluorescence values calculated from 65 neurons under Ca^2+^-free conditions (grey). For comparison, control data from 229 cells are shown (black, see also Fig. 2B), unmasking proportionate contributions of intra- and extracellular calcium sources to the NPSR1-mediated calcium signal. (C) Statistical analysis of the data shown in (B). The response amplitude has been integrated over time and averaged for different experimental conditions as indicated. (D) Mean fluorescence values from 47 neurons with calcium free extracellular solution and NPS applied as indicated. Error bars have been omitted for clarification. Calcium mobilization from intracellular stores is a prerequisite for generation of the slow, extracellular component of NPSR1-mediated calcium signals. (E) Statistical analysis of the data shown in (D). The response amplitude has been integrated over time and averaged for different experimental conditions as indicated. NPS was used at 500 nM in all experiments.

The biphasic time course of NPSR1-mediated calcium signals with a fast and a slow component suggested two major calcium routes. Therefore, the contribution of calcium influx via the plasma membrane was tested in a nominally calcium-free solution. We found the amplitude of the initial fast component of the NPS-evoked calcium signal not to be significantly altered under calcium-free conditions, with values of 3.6 ± 2.9 and 2.9 ± 1.9, respectively (t-test: p = 0.1054). In contrast, the duration and amplitude of the slow component was drastically reduced (Fig. 6A,B). This indicates that the initial rapid component in the NPSR1-mediated calcium signal could be attributed to mobilization of calcium from the ER, while the long lasting slow component was caused by an influx of extracellular Ca^2+^. The response amplitude was integrated over time for the slow component of the calcium signal in order to quantify the Ca^2+^ influx. The integral was significantly decreased from 36.6 ± 8.3 x 10^4^ (arbitrary units) under control conditions to 23.4 ± 3.9 x 10^4^ in Ca^2+^-free solution (Fig. 6C, t-test: p < 0.0001).

These results suggested that calcium entry pathways located in the plasma membrane largely contributed to the second, slower component of NPSR1-evoked increases in cytosolic Ca^2+^. In most cells, calcium release from the ER and the according fall in ER-luminal Ca^2+^ concentration subsequently activates calcium channels in the cell membrane in a process called store-operated calcium entry (SOCE) [35]. Next, we tested our hypothesis that NPSR activation induces SOCE in a downstream process. To further discriminate contributions of intra- and extracellular calcium sources, we applied NPS in a nominally calcium-free solution to induce the fast, intracellular component of the NPSR1 dependent calcium signal. When the fluorescence was back to baseline, we perfused the cells with standard extracellular solution containing Ca^2+^ to initiate SOCE (Fig. 6D). To quantify the resulting calcium influx, we integrated the response amplitude over time after re-addition of Ca^2+^ (Fig. 6E). We found that the integral was drastically reduced from 14.8 ± 7.2 x 10^4^ (arbitrary units) for a first application of NPS under calcium-free conditions to 2.1 ± 3.2 x 10^4^ for a control application of Ca^2+^-free extracellular solution without NPS. After applying NPS under Ca^2+^-free conditions once again, the integral was significantly increased to 5.9 ± 4.9 x 10^4^ (n = 47 cells, ANOVA: F_(2, 138)_ = 67.16, p < 0.0001).

**Fig 7:**
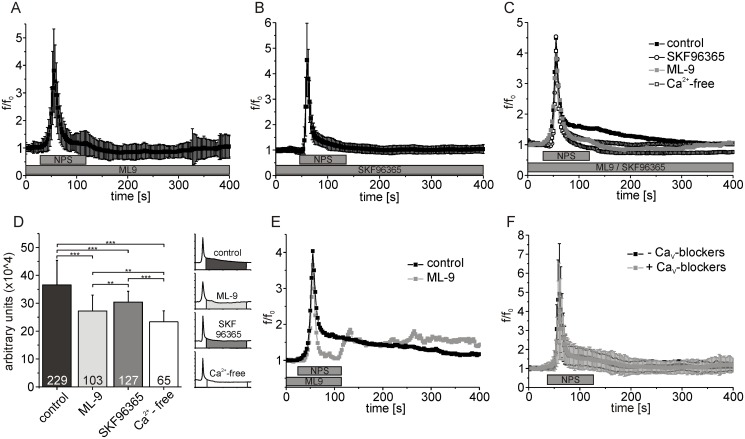
SOCE blockers inhibit the generation of the slow, extracellular component of NPSR1-mediated calcium signals. (A) Mean fluorescence values calculated from 103 NPSR1-expressing neurons recorded in the presence of ML-9 [50 μM]. (B) Mean fluorescence values of 127 cells treated with SKF96365 [15 μM]. (C) Overlay of mean fluorescence values from 229 cells recorded under control conditions, i.e. extracellular solution supplemented with 3 mM CaCl_2_ (black, see also Fig. 2B), 103 neurons recorded in presence of ML-9 (grey, see also Fig. 7A), 127 cells treated with SKF96365 (empty circles, see also Fig. 7B), and 65 neurons recorded under calcium-depleted conditions (empty squares, see Fig. 6B). (D) Statistical analysis of the data shown in (C). The response amplitude has been integrated over time and averaged for different experimental conditions as indicated. (E) Overlay of mean fluorescence of 15 cells treated with NPS and ML-9 [100μM] as indicated (grey) and 207 cells measured under control conditions (black). (F) Normalized mean fluorescence values from 148 neurons expressing NPSR1 from a first application of NPS and a consecutive second application of NPS in presence of Mibefradil [4 μM], Nifedipine [5 μM] and Conotoxin MVIIC [250 nM]. Standard deviations have been omitted for clarification in (C) and (E). Calcium signals were evoked with 500 nM NPS as indicated.

In previous studies, ML-9 and SKF96365 have been shown to inhibit SOCE in non-excitable cells [36–38] as well as in neurons [39]. In the present study, ML-9 [50 μM] and SKF96365 [15μM] consistently reduced the duration and amplitude of the slow component of NPSR1-evoked calcium signals (Fig. 7A,B), similar to the result obtained using calcium-free extracellular solution (Fig. 7C). ML-9 significantly decreased the integral of the response amplitude of the slow component from 36.6 ± 8.3 x 10^4^ (arbitrary units) for control conditions to 27.2 ± 5.7 x 10^4^ in experiments with the drug (Fig. 7D, ANOVA: F_(3, 520)_ = 87.46, p < 0.0001). In presence of SKF96365, a reduction to 30.42 ± 3.8 x 10^4^ could be observed (Fig. 7D). When NPS and ML-9 were washed out in parallel, we found that the fluorescence intensity was increasing to the same level as observed under control conditions (Fig. 7E). In summary, ML-9 and SKF96365 significantly reduced NPSR1-evoked influx of extracellular calcium.

Finally, a possible contribution of voltage activated calcium channels (Ca_v_) to the NPS-evoked calcium response was tested. Using a combination of Ca_v_ antagonists (Mibefradil [4 μM], Nifedipine [5 μM] and Conotoxin MVIIC [250 nM]), we found the NPS-triggered calcium signal unaffected compared to control in 148 neurons (Fig. 7F). The integral of the response amplitude during the recovery phase did not change significantly (36.2 ± 7.5 x 10^4^ (arbitrary units) for control conditions and 37.4 ± 7.8 x 10^4^ in presence of the Ca_v_ blockers, t-test: p = 0.2048).

## Discussion

In this study, we used heterologous expression of the human NPS receptor in mouse neurons *in vitro* in order to study intracellular mechanisms triggered by NPSR1 activation. We provide evidence that (i) NPSR1 can be functionally expressed in mouse neurons, (ii) application of NPS leads to calcium release from the endoplasmic reticulum via IP_3_ and ryanodine receptors in NPSR1 expressing neurons and (iii) these calcium release mechanisms in turn activate SOCE, thereby inducing influx of extracellular calcium.

Human NPSR1-mCherry expressed after adenoviral transfection displayed punctual localization across the soma and dendrites of transfected neurons. A similar distribution has been shown for the G-protein coupled receptors (GPCRs) neurokinin1 and somatostatin receptor using immunohistochemistry [40,41]. In surface biotinylation assays, we found that a fraction of NPSR1-mCherry proteins was targeted to the plasma membrane. In immunoblots, NPSR1-mCherry appeared in two bands of 74 kDa and 67 kDa, resembling previously published findings where HA-tagged NPSR1 was identified in two bands of 52 kDa and 44 kDa [42]. It has been shown in the same study that the high MW band corresponds to the maturated fraction of NPS receptors. We detected predominantly the high MW form of NPSR1 in the plasma membrane fraction, indicating maturation along the secretory pathway for the membrane-integrated subset of expressed NPSR1-mCherry proteins. Moreover, calcium imaging revealed specific and reproducible responses to NPS application, proving that NPSR1-mCherry proteins were functionally active.

**Fig 8:**
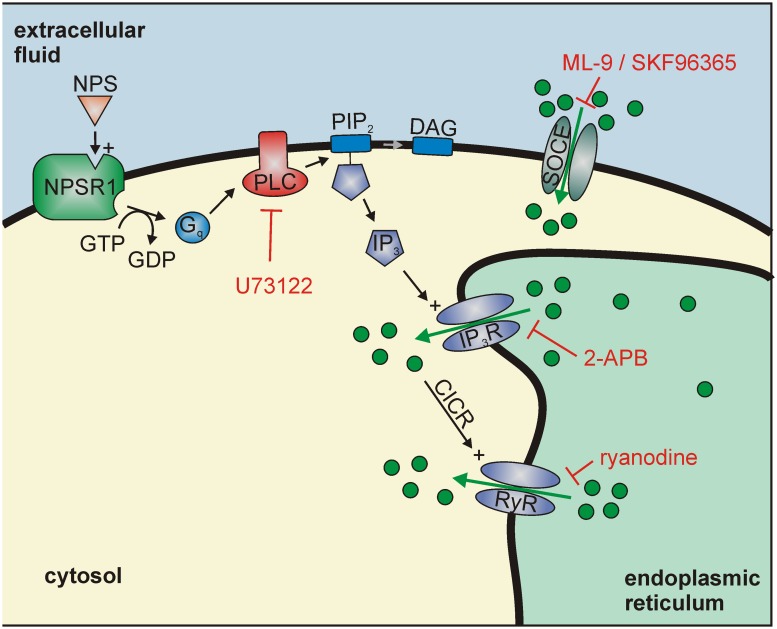
Model for the intracellular mechanisms underlying NPSR1 activation in cultured mouse hippocampus neurons. Calcium is released from the endoplasmic reticulum via IP_3_ and ryanodine receptors, which can be blocked by U73122, 2-APB and ryanodine, respectively. This decrease in the ER calcium content activates store-operated calcium entry (SOCE), which can be visualized by using Ca^2+^-free extracellular solution, the underlying signal cascade can be blocked by ML-9 and SKF96365.

In previous *in vitro* studies, NPSR1 has been expressed in HEK293 or CHO cells [1,10,24,25,43]. These studies focused on comparing NPS and NPSR1 variants with respect to their Gα_q_ and Gα_s_ activity, i.e. their efficiency to induce intracellular calcium responses and cAMP production upon receptor stimulation, analyzing population responses from plate readers instead of confocal single-cell calcium imaging. The results of the present study extend these previous findings by providing a detailed characterization of the molecular signaling pathways and mechanisms underlying NPSR1-mediated Ca^2+^ mobilization in individual neurons with single-cell resolution. The cultured hippocampal neurons used represent a well-established and well-characterized expression system. They seem particularly suited for our study since untransfected hippocampal neurons proved to be non-responsive to NPS in our control experiments. Based upon our results, a model of NPSR1-coupled signaling pathways can be constructed (Fig. 8), which incorporates NPSR1-dependent Ca^2+^ release from the ER by IP_3_ receptor activation. The findings that no significant rise in [Ca^2+^]_cyt_ could be detected upon application of NPS in the presence of the PLC blocker U73122 [34] or the IP_3_R antagonist 2-APB [33] shows that IP_3_ generation and IP_3_R activation are essential for the generation of NPSR1-mediated calcium signals, and exclude a significant contribution of other calcium channels directly activated following NPSR1 stimulation. Moreover, these results indicate that the proposed Gα_s_ activity of NPSR1 does not take part in Ca^2+^ signal generation via a cAMP-dependent pathway. It is known that 2-APB not only inhibits IP_3_ receptors but also modulates several members of the transient receptor potential channel family [44], which are located in the plasma membrane. Therefore it is important to point out that we can clearly associate the effect of the drug to calcium release from the ER because NPSR1-mediated calcium mobilization was abolished when neurons have been treated with CPA, i.e. when the ER was devoid of calcium. Moreover, calcium release was still present in experiments where Ca^2+^-free extracellular solution was used that was supplemented with 5 mM EGTA.

In our experiments, blockade of RyRs by ryanodine significantly reduced the amplitude of the NPS-evoked calcium response, unmasking a second calcium release pathway from the endoplasmic reticulum. When IP_3_Rs were blocked, no NPS-dependent elevation in cytosolic calcium could be detected. Moreover, the amplitude of NPSR1-mediated calcium signals was not significantly decreased when calcium-free extracellular solution was used. This shows that NPSR1-dependent activation of RyRs strictly requires calcium release from the ER, a process previously described in neurons which is commonly referred to as calcium-induced calcium release (CICR) [45]. Taken together, calcium release from the ER upon IP_3_ receptor activation was amplified by subsequent activation of ryanodine receptors.

In many cell types, Gα_q-_mediated release of calcium from the endoplasmic reticulum is followed by the activation of calcium channels at the plasma membrane, known as store-operated calcium entry (SOCE) [46]. We used a nominally Ca^2+^-free extracellular solution to isolate these calcium routes and found the fast component of the NPSR1-mediated calcium signal unaffected while the slow component was abolished under these conditions. Re-addition of Ca^2+^ restored the slow, extracellular component of the NPS-induced Ca^2+^ signal while re-addition of Ca^2+^ following application of Ca^2+^-free solution without co-application of NPS did not induce influx of extracellular Ca^2+^. These results show that Ca^2+^ mobilization from intracellular stores, i.e. a decrease of the ER calcium content, was a prerequisite for generation of the slow component of NPSR1-mediated Ca^2+^ signals, strongly suggesting that NPSR1 stimulation specifically activates SOCE in a downstream process.

Store-operated calcium channels (SOCs) responsible for SOCE are known to be composed by the calcium release-activated calcium modulator 1 (ORAI) [47] and members of the transient receptor potential type c (TRPC) channel families [48]. Channel-activation is induced by interaction with the ER calcium sensor stromal interaction molecule (STIM) [48,49]. In previous studies, ML-9 has been shown to block interaction of STIM and ORAI, thereby inhibiting SOCE in non-excitable cells [38] as well as in neurons [39]. SKF96365 has been shown to inhibit STIM-mediated SOCE in HeLa cells overexpressing STIM1 [50]. In our experiments, Ca^2+^ signals evoked by NPS application in the presence of ML-9 or SKF96365 were similar to those recorded under calcium-free conditions, abolishing calcium influx from the extracellular solution. An obvious explanation is that these drugs blocked signal transmission from the ER lumen to the plasma membrane via STIM, thereby inhibiting SOCE. In case of ML-9, washout of the drug increased the cytosolic calcium content to a level similar to control conditions, presumably because the ML-9-blocked signal transmission from the ER lumen to the plasma membrane via STIM was restored, resulting in SOC activation and Ca^2+^ influx from the extracellular solution. However, the question whether NPSR1-mediated SOCE comprises activation of ORAI or TRPC channels remains to be elucidated.

The NPS system is known as a key player in the modulation of fear and anxiety in both humans and rodents [5,21]. The question arises how the results presented herein are connected to neuronal circuitry in fear related brain regions. In our previous study, we found that activation of NPS receptors increased glutamatergic transmission from principal neurons of the lateral amygdala to GABAergic paracapsular intercalated cells [5]. Presumably, NPSR1-mediated calcium mobilization up-regulates neurotransmitter release and thereby enhances synaptic transmission. However, NPSR1-triggered calcium release can also be envisioned to induce long term modulation of synaptic plasticity: It is well known that the intracellular calcium concentration in synaptic terminals also takes part in the early phase of long term potentiation (LTP) via CaM kinase or protein kinase c dependent phosphorylation, which in turn enhances receptor recruitment to the postsynaptic membrane or modulates receptor activity directly (reviewed in [51]).

In human studies, it has been shown that a single-nucleotide polymorphism (A>T) of the *NPSR1* gene (rs324981) that codes for an amino acid exchange at position 107 (A107I) is more frequent in panic disorder patients [19–21]. Moreover this T-allele promotes over-interpretation of fear reactions in humans and ultimately leads to an increase of activity in the dorso-medial prefrontal cortex and the dorsal-anterior cingulate cortex, brain regions consistently linked with fear-conditioning paradigms [22]. A more recent study links the AA-phenotype to environment-depending anxiety and affective disorders, depressiveness and suicidal behavior in females [52]. The viral expression system we established allows for the characterization of individual components involved in NPSR1-mediated calcium signaling. It paves the way for comparative studies that will expand our understanding of the consequences of *NPSR1* polymorphisms at the molecular level and, considering the proposed clinical background of NPSR1, might provide new insights in the intracellular mechanisms involved in the generation of anxiety and anxiety disorders.
